# Effect of high carbohydrate high fat diet on hippocampal neurovascular coupling in a rat model of Alzheimer's Disease

**DOI:** 10.1002/alz70856_106905

**Published:** 2026-01-08

**Authors:** Dustin Loren V Almanza, Andrea Trevisiol, Margaret M Koletar, Mary Hill, JoAnne McLaurin, Bojana Stefanovic, Greg Stanisz

**Affiliations:** ^1^ Sunnybrook Research Institute, Toronto, ON, Canada; ^2^ University of Toronto, Toronto, ON, Canada; ^3^ Sunnybrook Health Sciences Centre, Toronto, ON, Canada; ^4^ Biological Sciences, Sunnybrook Research Institute, Toronto, ON, Canada; ^5^ Medical Biophysics, University of Toronto, Toronto, ON, Canada; ^6^ Physical Sciences, Sunnybrook Research Institute, Toronto, ON, Canada

## Abstract

**Background:**

Interaction of Alzheimer's Disease (AD) pathology and vascular comorbidities is difficult to study in humans due to slow course of AD and high prevalence of patients with mixed pathologies. Vascular comorbidities (e.g., with obesity) are highly prevalent and increase the risk of developing dementia. There is thus a pressing need to examine experimental AD models incorporating obesity and to establish sensitive and translational imaging assays therein.

**Method:**

TgF344‐AD (TgAD) rats and their non‐transgenic (nTg) littermates were given three months *ad lib* access to high carbohydrate high fat (HCHF) food items, so as to imitate the highly palatable foods consumed in the obesogenic population, before MR imaging them at 12 months of age (established AD). Pseudo continuous arterial spin labeling (pCASL) MRI was utilized to establish an assay of hippocampal neurovascular compromise and its sensitivity to AD and its interaction with obesity.

**Result:**

In CHOW‐fed cohorts, hippocampal functional hyperemia (CBF change and volume of activation) in response to electrical stimulation of the forepaw was attenuated in TgAD rats (CBF change: 7 ± 14 ml/100g/min and volume of activation: 0.03 ± 0.08) relative to nTg rats (CBF change: 49 ± 21 ml/100g/min, *p* = 0.029 and volume of activation: 0.34 ± 0.14, *p* <0.001). In contrast, the functional hyperemia was enhanced with HCHF diet in TgAD rats (CBF change: 60 ± 26 ml/100g/min, *p* <0.001 and volume of activation: 0.25 ± 0.15, *p* <0.001).

**Conclusion:**

The observed rescue of functional hyperemia with HCHF in established AD, but not in normal aging, is speculated to result from metabolically dysregulated AD brain profiting from calorie‐dense food consumption. This work demonstrates a non‐invasive neuroimaging assay to assess hippocampal neurovascular function in AD and its vascular comorbidities, offering high translational potential. This assay affords ease of delivering somatosensory stimuli and limited effect of aging and neurodegeneration on somatosensation, making somatosensory protocol a particularly easy one to deploy in patients.

